# Changes in the use practitioner-based complementary and alternative medicine over time in Canada: Cohort and period effects

**DOI:** 10.1371/journal.pone.0177307

**Published:** 2017-05-11

**Authors:** Mayilee Canizares, Sheilah Hogg-Johnson, Monique A. M. Gignac, Richard H. Glazier, Elizabeth M. Badley

**Affiliations:** 1 Institute of Medical Science, University of Toronto, Toronto, Ontario, Canada; 2 Krembil Research Institute, University Health Network, Toronto, Ontario, Canada; 3 Dalla Lana School of Public Health, University of Toronto, Toronto, Ontario, Canada; 4 Institute for Work and Health, Toronto, Ontario, Canada; 5 Institute for Clinical Evaluative Sciences, Toronto, Ontario, Canada; 6 Department of Family and Community Medicine, University of Toronto, Toronto, Ontario, Canada; 7 Department of Family and Community Medicine, St. Michael’s Hospital, Toronto, Ontario, Canada; McMaster University, CANADA

## Abstract

**Background:**

The use of complementary and alternative medicine (CAM) is growing. However the factors contributing to changes over time and to birth cohort differences in CAM use are not well understood.

**Setting:**

We used data from 10186 participants, who were aged 20–69 years at the first cycle of data collection in the longitudinal component of the Canadian National Population Health Survey (1994/95-2010/11). We examined chiropractic and other practitioner-based CAM use with a focus on five birth cohorts: pre-World War II (born 1925–1934); World War II (born 1935–1944); older baby boomers (born 1945–1954); younger baby boomers (born 1955–1964); and Gen Xers (born 1965–1974). The survey collected data every two years on predisposing (e.g., sex, education), enabling (e.g., income), behavior-related factors (e.g., obesity), need (e.g., chronic conditions), and use of conventional care (primary care and specialists).

**Results:**

The findings suggest that, at corresponding ages, more recent cohorts reported greater CAM (OR = 25.9, 95% CI: 20.0; 33.6 for Gen Xers vs. pre-World War) and chiropractic use than their predecessors (OR = 2.2, 95% CI: 1.7; 2.8 for Gen Xers vs. pre-World War). There was also a secular trend of increasing CAM use, but not chiropractic use, over time (period effect) across all ages. Factors associated with cohort differences were different for CAM and chiropractic use. Cohort differences in CAM use were partially related to a period effect of increasing CAM use over time across all ages while cohort differences in chiropractic use were related to the higher prevalence of chronic conditions among recent cohorts. The use of conventional care was positively related to greater CAM use (OR = 1.8, 95% CI: 1.6; 2.0) and chiropractic use (OR = 1.2, 95% CI: 1.1; 1.4) but did not contribute to changes over time or to cohort differences in CAM and chiropractic use.

**Conclusion:**

The higher CAM use over time and in recent cohorts could reflect how recent generations are approaching their healthcare needs by expanding conventional care to include CAM therapies and practice for treatment and health promotion. The findings also underscore the importance of doctors discussing CAM use with their patients.

## Introduction

Conventional or mainstream medicine continues to be the main source of healthcare in Canada and elsewhere. However, a significant number of people choose complementary and alternative medicine (CAM) for wellness and/or treatment [1, 2]. The increasing demand for CAM may reflect a diversification of preferences for different types of healthcare services and an increasing emphasis on health promotion and self-care by the public [3]. For example, studies show that while many adults use CAM therapies to treat specific symptoms such as chronic pain, others also report using CAM for general health maintenance [4–6]. Therefore, the growing interest in CAM raises questions about the patterns of CAM use over time in the context of use of conventional medicine in the population. Understanding the changes in patterns of CAM and conventional care use has important implications for planning and improving the healthcare system as well as for medical education.

Age has been found to be strongly related to CAM use, but studies show an inconsistent pattern. Some studies suggest CAM use peaks at middle age [7–10] while others show that CAM use increases with increasing age [11, 12]. It is not clear if these findings reflect true age effects or if they are related to birth cohort effects. Cohort effects arise from differences in the experiences of groups born and growing up in different time periods. These may be differences that are unique to a particular birth cohort or that accumulate over the lifetime. Only two studies have examined cohort differences in CAM use [13, 14]. Both reported greater CAM use in more recent cohorts, but used cross-sectional data and could not distinguish cohort effects from age and period effects (secular changes over time). Period effects are changes in CAM use across all age groups resulting from widespread societal changes or from events that took place at a particular point in time. Changes in government policies are often cited as examples of period effects.

Findings from studies examining changes over time in CAM use are not consistent across CAM therapies and practices. For example, data from the U.S. on national estimates of CAM use for 2002, 2007, and 2012 found large variability across types of CAM used and over time [15]. The study found an increased use of acupuncture and homeopathy over time, but no significance change in chiropractic use. A Canadian study showed that use of publicly subsidized chiropractic services by adults over the age of 50 decreased over the decade of the 1990s in one Canadian province [16]. Several hypotheses related to period differences have been put forward to explain why CAM use may be growing, including the rise of the consumer movement in healthcare since the 1970’s and the growing use of the Internet [17–19]. The consumer movement has empowered individuals and encouraged them to take a proactive role towards healthcare decisions and selection of services [18, 20, 21]. Additionally, as health information becomes more readily available online, more individuals are seeking and finding information about CAM treatments, which they may incorporate into their general healthcare practices [19].

The aging of the population has been proposed as another possible explanation for variations in CAM use over time. Notably, age effects alone do not appear to explain these changes since analyses adjusting for age continue to indicate variability across time [22, 23]. These variations may also be related to changes in other factors. For example, cross-sectional studies have found that CAM use is greater among those with high income and/or educational levels [2, 7, 9, 24]. Yet, the few studies that have controlled for these factors while comparing CAM use across periods of time have found that changes in income and education were not associated with time trends in CAM use [23]. Health variables are also important. Studies consistently indicate that chronic conditions and pain are significantly related to CAM use [7, 13, 25–27]. Therefore, it is reasonable to hypothesize that the growing number of people living with chronic conditions may also underlie the growing trend in CAM use [28–30]. Lastly, the use of CAM in relation to conventional care has also been examined with inconsistent findings. Some studies suggest that conventional care users supplement their care with CAM services [31, 32]. Others have suggested that patients having difficulties accessing conventional care turn to CAM to meet their healthcare needs [33, 34]. No study, however, has examined changes in CAM use over time in the context of changing patterns of need for care and of conventional care use.

We drew on 16 years of longitudinal population data to examine variations in CAM use from 1994 to 2011 We focused on five birth cohorts of Canadians: pre-World War II (born 1925–1934), World War II (born 1935–1944), older baby boomers (born 1945–1954), younger baby boomers (born 1955–1964), and Generation X (Gen Xers, born 1965–1974). We also controlled for other factors associated with CAM use that have been reported in the literature (e.g. chronic conditions, pain). Our goals were to determine 1) whether, in addition to age effects, there were birth cohort and/or period effects in CAM use, and 2) whether changes in need for care and changes in the use of conventional medicine contributed to any cohort and/or period effects, controlling for other factors. We hypothesized that changes in CAM use over time were, at least partially, related to cohort and period effects, independent of changes over time in the factors predicting CAM use.

## Materials and methods

### Canadian National Population Health Survey

We used data from the longitudinal component of the Canadian National Population Health Survey (NPHS) spanning 16 years (1994–2011) [35]. The target population of the NPHS included household residents in the ten Canadian provinces in 1994/1995. The survey excluded persons living on Indian Reserves and Crown Lands, residents of health institutions, full-time members of the Canadian Forces Bases and some remote areas in Ontario and Québec. The survey used a complex sampling design with a multi-stage stratified and cluster selection (geographic and/or socio-economic strata, geographic clusters, and then dwellings within each cluster). The NPHS retained individuals who moved to long-term care institutions and those who died over the course of the survey. The death of a respondent was confirmed against the Canadian Vital Statistics Database, and the cause and date of death were captured. More details on the NPHS sampling plan and survey questions is available from Statistics Canada [35].We restricted the sample to 10186 individuals aged 20–69 years in 1994 who provided at least three cycles of data.

This paper is based on secondary analyses of data collected by Statistics Canada; as such we did not obtained direct consent from the survey participants. However, participation in the survey was voluntary and respondents consented that their data may be used by third parties upon approval from Statistics Canada. In addition, the University of Toronto Ethics Committee approved the study.

### Measures

#### CAM use

CAM use was defined as consulting with any of the following CAM practitioners (Yes/No) in the past 12 months: massage therapist, acupuncturist, homeopath or naturopath, Feldenkrais or Alexander teacher, relaxation therapist, biofeedback teacher, rolfer, herbalist, reflexologist, spiritual healer, or religious healer. As in other studies we also included chiropractors [36]. Because chiropractors exhibit characteristics of both conventional medicine and CAM [37, 38], we examined chiropractic use separately from other CAM use. Chiropractic use was derived from a separate question: “In the past 12 months, how many times have you seen or talked on the telephone with a chiropractor about your physical, emotional or mental health?” Chiropractic use was defined as reporting 1 or more visits.

#### Age, period, and cohort

We used participants’ date of birth to calculate age for each cycle and to allocate participants to the five birth cohorts previously noted. The year of the interview was used as an indicator of period.

#### Need for care

We used two factors to assess need for care: chronic conditions and pain that prevents activities. The NPHS collected data on up to 17 individual chronic conditions that had been diagnosed by a healthcare professional: arthritis, back problems, asthma, allergies (excluding food allergies), bronchitis, emphysema, diabetes, high blood pressure, heart conditions, stroke, cancer, ulcers, urinary incontinency, dementia, migraine, glaucoma, and cataracts. We calculated the number of chronic conditions and grouped them as: none, 1, and 2+. For the variable “pain that prevents activities” responses were grouped as: no pain/pain does not prevent activity or pain prevents activity (few/sometimes/always).

#### Use of conventional care

At each survey cycle participants reported whether they contacted primary care physicians/general practitioners (PCP) or specialists (excluding eye care) in the 12 months prior to their interview. An indicator combining use of PCPs and of specialists was created: visited both, only PCP, only specialists, none.

#### Other predictors of CAM use

We included other factors grouped as predisposing, enabling and behavior-related that previously have been found to be associated with CAM use [7]: predisposing (sex and education), enabling (household income and having a regular source of care), and behavior-related (obesity, smoking status, physical activity, and sedentary lifestyle). Education was measured as years of schooling and was grouped for analyses as: <12 years, 12–15 years, and 16+ years. At each cycle, participants reported if they had a regular doctor. Household income was categorized into quartiles of the distribution within each survey year and a separate category representing unknown values was retained for analyses. Obesity was ascertained by using body mass index (BMI) categorized as: underweight (<18.5), normal weight (18.5–24.9), overweight (25.0–29.9), moderate obese (30.0–34.9), and severe obese (≥35.0). Smoking status was assessed by a Statistics Canada derived variable which grouped participants as current smoker, former smoker, and non-smoker (those who never smoked) [35]. Responses to a series of questions about participation in leisure time physical activities such as, walking for exercise, running, gardening, etc. combined with data on walking or bicycling for commuting were used to group individuals as physically active (during leisure time or active commuting) vs. inactive. Lastly, sedentary lifestyle was defined as those who reported that they “usually sit during the day and don’t walk around very much.”

### Statistical analysis

There is ongoing debate as to the best way to examine the unique effects of age, period, and cohort [39–41]. Because age, period and cohort are linearly related, the linear effects of the three factors cannot be modeled simultaneous without imposing restrictions on at least one of the parameters. For this study, we conceptualized the models within a multilevel framework [42, 43]. We estimated age and cohort as fixed effects with period as a random effect. We started with a model unadjusted by period (Model 1). This was a two-level model where repeated observations were nested within individuals, and age and cohort effects were estimated as fixed effects. We then extended this model by adding another level to account for variability across periods of times (Model 2). This was a hierarchical age-period-cohort (HAPC) model in which repeated observations were nested within individuals and individuals were nested within time periods. In subsequent models we added need for care and use of conventional care while adjusting for the other predictors of CAM previously listed. In addition to examining the effect of need for care and use of conventional care on CAM use, we also examined whether these factors affected the cohort, age, and period estimates.

We used the SAS/STAT software for all data analyses and the GLIMMIX procedure to estimate the HAPC model [44]. The procedure uses maximum likelihood estimators that adjust for non-response assuming the data are missing at random. It also uses all available data for incomplete cases [44]. Although the NPHS uses weights to compensate for the complex multistage sample design, the results of this paper are based on un-weighted analyses. The reason for this is that the HAPC model cannot incorporate sampling weights at cross-classified levels. We centered age at 39 years (the mean of the distribution for the five cohorts at baseline (1994/95)). We used Wald tests to assess the significance of the variables.

#### Sub-analyses

About 39% of eligible participants died or dropped-out before the end of the study. To examine the effect of attrition in our findings we compared our main results with the results of two additional analyses: 1) including indicator variables identifying participants who dropped-out or died before the end of the study in all models; and 2) analyses with a restricted sample of participants with complete data in the nine cycles.

Previous studies have suggested that analyses grouping CAM practitioners have the potential of missing differing patterns of use across practitioners [45]. We, therefore, repeated our analyses for the CAM practitioners with >1% of use: massage therapy, acupuncture, and homeopathy/naturopathy.

We also repeated the analyses to examine the contribution of specific chronic conditions to the results. We chose the conditions that have been reported in the literature to be associated with CAM use. These conditions were: back pain, arthritis, respiratory (asthma, allergies, bronchitis, or emphysema), migraine, diabetes, high blood pressure, cardiovascular (heart conditions or stroke), cancer, and other (ulcers, urinary incontinency, dementia, glaucoma, cataracts).

Nahin et al [46] found that about 25% of individuals who do not use conventional care use CAM in subsequent years. We, therefore, fitted the final model for CAM use with an additional variable indicating the use of conventional medicine in the previous cycle of data collection. This way we could examined whether those not using conventional care had increased odds of using CAM in the following year.

## Results

### Descriptive

There were 10186 participants with at least three years of data (13.6% in the pre-World War II cohort, 15.7% in the World War II, 21.6% in the older baby boomer, 27.3% in the younger baby boomer, and 21.8% in the Generation X). Overall, 10.0% of the initial sample died and 27.3% dropped-out during follow-up. Between 1994/95 and 2010/11, CAM use increased from 4.8% to 11.2%. In contrast, overall chiropractic use remained virtually constant (9.0% in 1994/95 vs. 10.2% in 2010/11, respectively). Similar patterns were seen in all birth cohorts. Chiropractors were the most common type of practitioner consulted across all cohorts followed by massage therapists (Table 1). Generally, users of all types of CAM practitioners had higher education and/or income. They were less likely to be current smokers, were more physically active, and more likely to have a sedentary lifestyle. Obese individuals were less likely to consult with other CAM practitioners and more likely to consult with chiropractors. CAM users also had more chronic conditions and a higher proportion reported pain. In addition, CAM users reported higher use of conventional care (visits to PCPs and/or specialists) than non CAM users (Table 2).

**Table 1:** Use (%) of practitioners-based complementary and alternative medicine in 1994/95 and 2010/11 by birth cohort. Canadian National Population Health Survey (NPHS), 1994–2011.

	ALL (1925–1974)		PRE-WORLD WAR II (1925–1934)		WORLD WAR II (1935–1944)		OLDER BABY BOOMER (1945–1954)		YOUNGER BABY BOOMER (1955–1964)		GENERATION X (1965–1974)	
	cycle 1: 1994/95	cycle 9: 2010/11	cycle1: 1994/95	cycle 9: 2010/11	cycle1: 1994/95	cycle 9: 2010/11	cycle1: 1994/95	cycle 9: 2010/11	cycle1: 1994/95	cycle 9: 2010/11	cycle1: 1994/95	cycle 9: 2010/11
*n*	10186	6562	1384	665	1596	1061	2205	1577	2778	1886	2223	1373
CAM use (all practitioners)	14.6	24.5	12.1	11.5	15.4	16.1	17.4	24.1	16.3	29.5	10.8	31.0
Chiropractors	10.7	13.4	9.5	8.4	11.8	9.0	13.0	13.8	11.2	15.7	8.2	15.6
CAM use ^a^ (other practitioners)	5.6	15.8	3.8	5.3	5.2	9.2	6.6	14.6	7.3	19.6	3.9	21.8
Massage therapist	2.4	7.6	1.0	3.5	3.1	5.6	3.2	9.1	4.0	14.2	2.3	15.6
Acupuncturist	0.8	2.4	1.2	1.5	0.8	2.5	1.0	3.4	1.0	3.6	0.3	3.8
Homeopath/ Naturopath	1.2	1.9	0.9	0.4	1.3	1.3	1.7	2.8	2.0	3.4	0.9	2.8

Abbreviations: CAM, Complementary and Alternative Medicine

^a^ Massage therapist, Acupuncturist, Homeopath/Naturopath, Feldenkrais or Alexander teacher, relaxation therapist, biofeedback teacher, rolfer, herbalist, reflexologist, spiritual healer, or religious healer

**Table 2:** Characteristics of users and non-users of CAM and chiropractic services. Canadian National Population Health Survey (NPHS), 1994–2011.

	CAM				Chiropractic			
	Cycle 1: 1994/95		Cycle 9: 2010/11		Cycle 1: 1994/95		Cycle 9: 2010/11	
	Users	Non-users	Users	Non-users	Users	Non-users	Users	Non-users
*n*	570	9616	1035	5362	1100	9086	882	5705
**Mean number of chronic conditions**	1.3	0.9	2.0	1.9	1.4	0.9	2.1	1.9
% with back pain	26.9	15.1	29.2	19.7	41.7	12.6	37.5	18.7
% with arthritis	15.1	13.1	29.2	30.8	17.6	12.6	31.2	30.5
% with migraine	12.1	8.3	13.5	8.5	11.3	8.2	11.8	8.5
% with respiratory	27.8	22.0	44.8	34.4	27.8	21.6	42.3	35.0
% with diabetes	3.5	2.5	5.4	10.3	2.9	2.5	7.1	10.0
% with high blood pressure	7.4	8.9	19.3	30.6	8.6	8.9	23.4	29.6
% with cancer	1.8	1.2	1.5	2.3	1.1	1.3	1.7	2.2
% with cardiovascular	4.2	3.1	5.8	9.5	3.0	3.6	7.8	9.0
% with other ^a^	17.8	13.2	29.5	31.2	16.0	13.2	28.5	31.3
% with no chronic conditions	34.7	47.6	21.1	22.7	37.0	22.2	19.4	22.9
**% with pain**	21.9	10.9	21.6	14.8	19.4	10.5	20.1	15.3
**Use of conventional care**								
% consulting with PCP	89.1	77.4	86.1	78.0	85.8	77.1	83.8	78.6
Mean visits to PCP	5.1	3.6	3.7	3.2	4.5	3.6	3.5	3.2
% consulting with specialists	40.0	25.8	37.2	31.9	30.6	26.1	35.0	32.4
Mean visits to specialists	1.8	0.9	1.2	1.0	1.3	1.0	1.0	1.0
**Other factors**								
*Predisposing*								
% women	70.4	53.1	71.5	53.1	54.7	54.0	56.7	55.9
Mean years of education	13.6	12.5	14.4	13.0	12.9	12.5	13.8	13.1
*Enabling*								
Mean household income ^b^	53.4	50.4	87.4	73.5	53.1	50.2	84.1	74.4
% with regular source of care	87.5	85.7	92.2	90.9	90.3	85.2	92.5	90.9
*Behavior-related factors*								
% obese (BMI ≥ 30.0)	45.3	52.0	57.6	67.6	51.5	46.0	66.1	66.1
Smoking status								
% Current smokers	30.7	34.8	12.6	20.1	29.5	35.1	15.1	19.6
% Former smokers	34.6	30.2	53.9	49.2	36.7	29.7	54.0	49.3
% physical inactive	52.0	54.3	34.9	40.6	49.5	54.7	36.8	40.1
% with sedentary lifestyle	25.3	19.5	31.1	24.8	21.0	19.7	24.9	25.9

Abbreviations: CAM, complementary and alternative medicine; BMI, Body Mass Index; PCP, Primary Care Physician.

^a^ ulcers, urinary incontinency, dementia, glaucoma, or cataracts. ^b^ in Canadian dollars and expressed in thousands.

### CAM use

#### Changes over time and birth cohort differences

**Fig 1:**
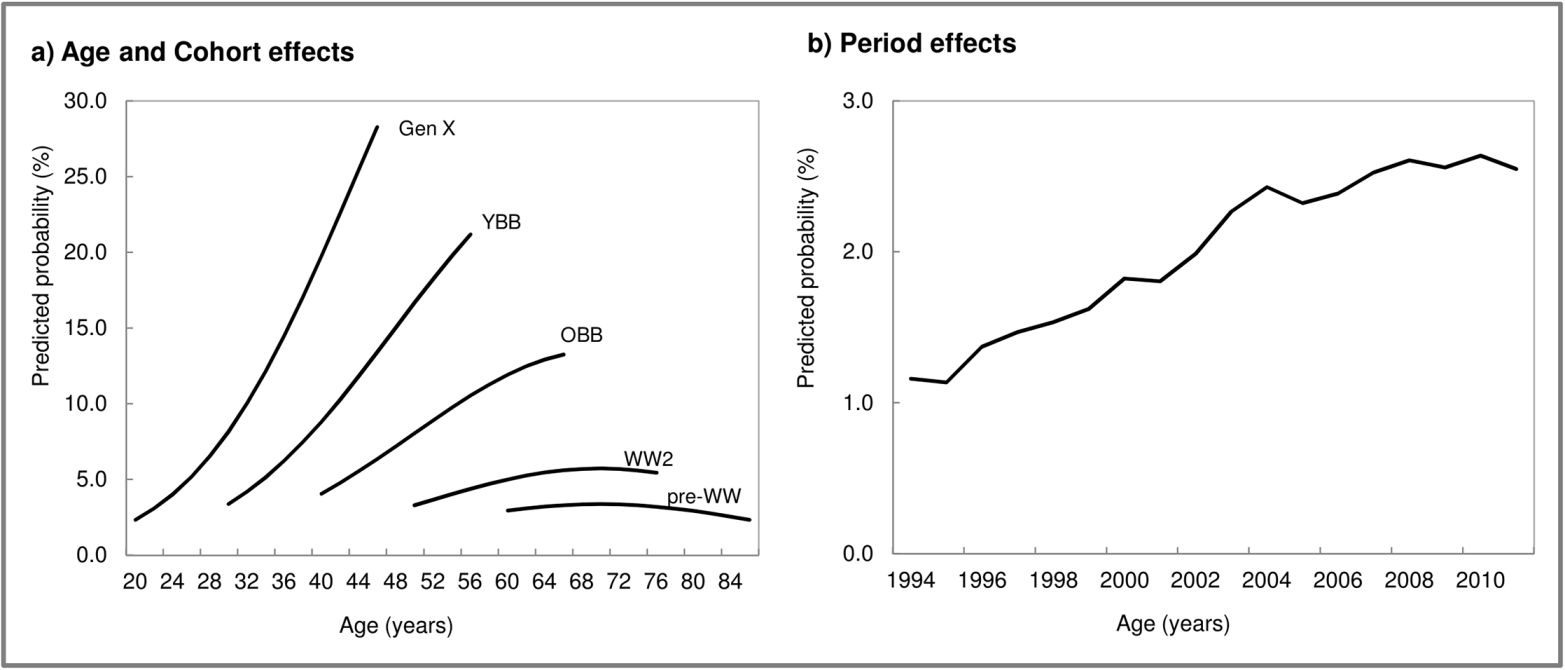
Age, period, and cohort effects for CAM use: Results from logistic growth models. **Canadian National Population Health Survey, 1994–2011.** Notes: CAM, Complementary and Alternative Medicine; GenX, Generation X; YBB, Younger Baby Boomer; OBB, Older Baby Boomer; WW2, World War II; pre-WW, pre-World War II. Values for a) are predictions from the fixed part of model 1 in Table 3 and values for b) are predictions from the solution of the random effects in model 2 in Table 3.

Table 3 presents the results of the modeling for CAM use. There were significant age differences in CAM use (Table 3, Model 1, Fig 1A). After accounting for the effects of aging, cohort differences were large and significant; that is, there was a trend of greater CAM use in each succeeding recent cohort, particularly for Gen Xers and baby boomers (Table 3, Model 1). Results from the model adjusting for period effects (Table 3, Model 2) indicated that there was significant variability in CAM use over this period of time. As illustrated in Fig 1B, there was a trend of increasing CAM use over the years irrespective of age and cohort. In addition, compared to the unadjusted model, the age and cohort effects were substantially reduced, although they remained significant. This suggests that broad societal changes were at least partially manifesting as increases in CAM use over the lifecourse.

**Table 3:** Results from logistic two-level growth model (1) and hierarchical age-period-cohort models (2–4) for CAM use. Canadian National Population Health Survey, 1994–2011.

	MODEL 1	MODEL 2	MODEL 3^a^	MODEL 4^a^
	OR (95% CI)	OR (95% CI)	OR (95% CI)	OR (95% CI)
**Fixed Effects**				
*Age and Cohort*				
Linear Age^b^	1.09 (1.09;1.10)***	1.02 (1.01;1.03)***	1.02 (1.00;1.03)*	1.02 (0.99;1.08)
Birth Cohort (Ref: Pre-World War)				
Generation X	25.90 (19.95;33.64)***	1.70 (1.02;3.00)***	1.78 (1.03;3.07)***	1.51 (1.14;2.00)**
Younger Baby Boomer	10.18 (8.00;12.95)***	1.34 (0.85;2.10)	1.36 (1.01;2.10)*	1.25 (1.01;1.61)*
Older Baby Boomer	4.46 (3.58; 5.56)***	1.17 (0.83;1.63)	1.13 (0.99;1.57)^†^	1.12 (0.99;1.40)
World War II	1.74 (1.42; 2.12)***	0.89 (0.70;1.13)	0.87 (0.69;1.10)	0.94 (0.77;1.15)
*Need for Healthcare*				
Chronic Conditions (Ref: None)				
2+			1.91 (1.75;2.08)***	1.79 (1.64;1.96)***
1			1.45 (1.34;1.58)***	1.40 (1.29;1.52)***
Pain Prevents Activity			1.91 (1.75;2.08)***	1.81 (1.66;1.98)***
*Conventional Care*				
Physician Visits (Ref: No visits)				
Both				1.78 (1.60;1.97)***
Primary Care Only				1.44 (1.30;1.58)***
Specialists Only				1.21 (1.00;1.47)*
**Random Effects** ^c^				
Individual	2.17 (2.06;2.28)***	2.14 (2.04;2.24)***	1.92 (1.82;2.02)***	1.92 (1.82;2.02)***
Period		0.20 (0.04;0.35)***	0.11 (0.01;0.20)***	0.11 (0.01;0.20)***

Abbreviations: OR, Odd Ratio; 95% CI, 95% Confidence Interval.

*** p<0.0001

** p<0.01

* p<0.05

^†^ p<0.1.

^a^ Models also included, predisposing, enabling, and behaviour-related factors. Full models are shown in S1 Table.

^b^ Age was centered at the mean of the distribution in 1994/95 (39 years). All models also included a quadratic age term.

^c^ Estimates are variances.

Model 2 was then extended to include predisposing, enabling, and behavior-related factors (S1 Table, Model 2a). CAM users were more likely to be women, have higher education/income, to not have a regular source of care, to be current smokers, have normal weight, be physically active, and to have a sedentary lifestyle. The estimates of the age and cohort effects remained significant in this model, but were slightly reduced. The model was further extended by adding need factors (Table 3, Model 3). (Only the estimates for age, cohort, need factors, and period are presented in the table with the full model presented in S1 Table, Model 3.) Cohort differences remained significant after accounting for need factors suggesting that there were cohort differences in CAM use over and above need for care. In addition, the estimate for the random effect for period was reduced but remained significant. This suggests that the trend of increasing chronic conditions over time partially underlies the growing CAM use. The inclusion of use of conventional care did not alter the age and cohort estimates (Table 3, Model 4).

#### Role of need for care and use of conventional care

As shown in Table 3 Model 4, chronic conditions and pain were strong predictors of CAM use. Those with two or more chronic conditions were more likely to use CAM than those with no chronic conditions (OR = 1.79, 95% CI (1.64; 1.96)). Similarly, those reporting pain were more likely to use CAM (OR = 1.81, 95% CI (1.66; 1.98)). The use of conventional care was also a significant and strong predictor of CAM use. The results indicate that CAM users were also users of conventional medicine: those consulting with primary care physicians and with specialists had higher odds of consulting with CAM practitioners.

### Chiropractic use

#### Changes over time and birth cohort differences

**Fig 2:**
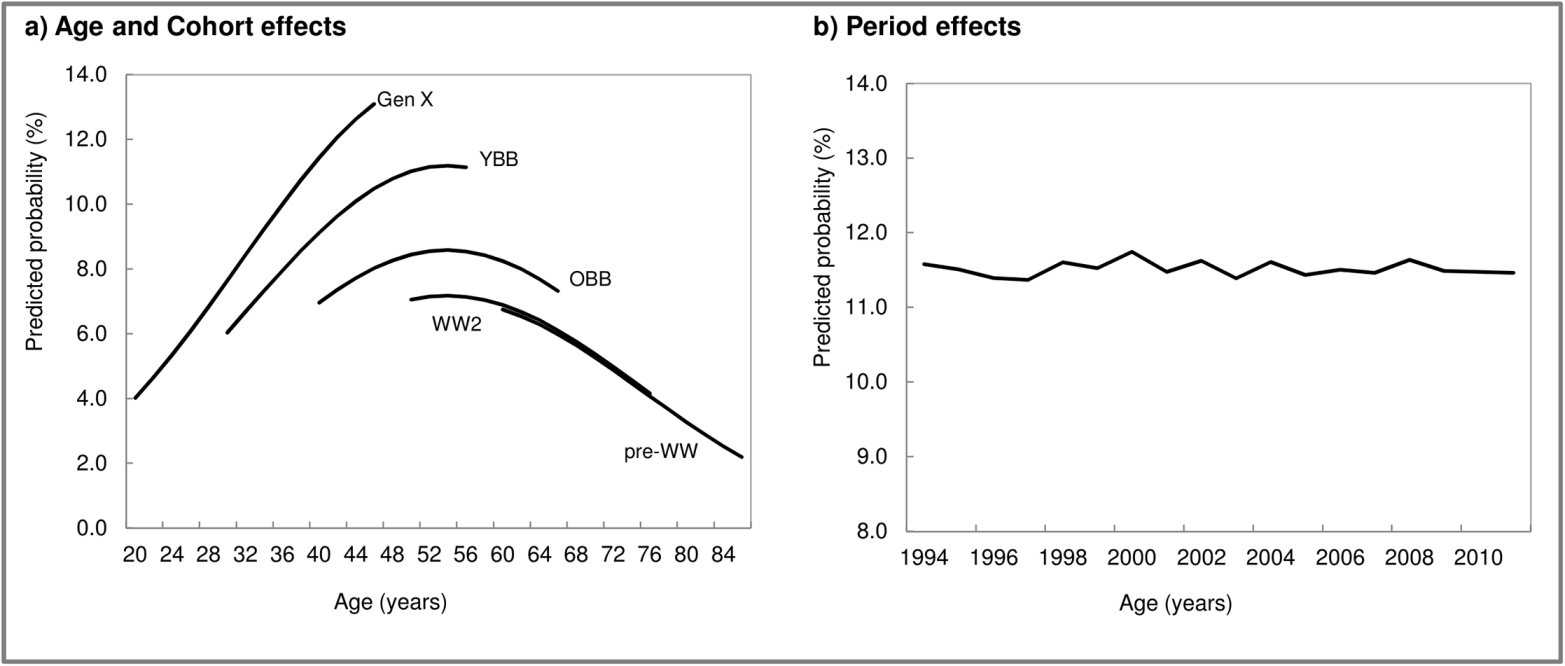
Age, period, and cohort effects for chiropractic use: Results from logistic growth models. **Canadian National Population Health Survey, 1994–2011.** Notes: GenX, Generation X; YBB, Younger Baby Boomer; OBB, Older Baby Boomer; WW2, World War II; pre-WW, pre-World War II. Values for a) are predictions from the fixed part of model 1 in Table 4 and values for b) are predictions from the solution of the random effects in model 2 in Table 4.

Results from the model unadjusted by period (Table 4, Model 1) showed that the age-trajectory of chiropractic use increased around middle age, then declined (Fig 2A). In addition to age effects, large and significant cohort differences were found (Table 4, Model 1, Fig 2A). Comparing cohorts at corresponding ages indicates that there was higher chiropractic use for Gen Xers, followed by younger boomers, and older boomers when compared to pre-boomers (World War II and pre-World War II cohorts). As shown by the estimate of the random effect for period, variability across years for chiropractor use was small and non-significant (Table 4, Model 2, Fig 2B). In addition, compared to the unadjusted model (Model 1), the age and cohort effects were virtually unchanged.

**Table 4:** Results from logistic two-level growth model (1) and hierarchical age-period-cohort models (2–4) for chiropractic use. Canadian National Population Health Survey, 1994–2011.

	MODEL 1	MODEL 2	MODEL 3^a^	MODEL 4^a^
	OR (95% CI)	OR (95% CI)	OR (95% CI)	OR (95% CI)
**Fixed Effects**				
*Age and Cohort*				
Linear Age^b^	1.04 (1.03;1.04)***	1.03 (1.03;1.04)***	1.01 (1.01;1.02)***	1.01 (1.01;1.02)***
Birth Cohort (Ref: Pre-World War)				
Generation X	2.16 (1.68;2.77)***	2.08 (1.58;2.75)***	1.12 (0.85;1.46)	1.15 (0.88;1.51)
Younger Baby Boomer	1.68 (1.34;2.11)***	1.64 (1.28;2.09)***	1.02 (0.80;1.29)	1.04 (0.82;1.32)
Older Baby Boomer	1.25 (1.01;1.53)***	1.23 (0.99;1.52)	0.87 (0.70;1.08)	0.88 (0.71;1.09)
World War II	1.03 (0.85;1.24)	1.02 (0.84;1.23)	0.83 (0.68;1.01)^†^	0.83 (0.69;1.01)^†^
*Need for Healthcare*				
Chronic Conditions (Ref: None)				
2+			2.37 (2.17;2.58)***	2.31 (2.11;2.52)***
1			1.61 (1.49;1.75)***	1.58 (1.46;1.72)***
Pain Prevents Activity			1.44 (1.32;1.58)***	1.43 (1.31;1.57)***
*Conventional Care*				
Physician Visits (Ref: No visits)				
Both				1.22 (1.11;1.35)***
Primary Care Only				1.25 (1.14;1.36)***
Specialists Only				1.01 (0.84;1.23)
**Random Effects**^c^				
Individual	2.66 (2.54;2.78)***	2.66 (2.54;2.78)***	2.59 (2.47;2.71)***	2.59 (2.47;2.71)***
Period		0.01(0.00;0.04)	0.01 (-0.02;0.03)	0.01 (-0.01;0.04)

Abbreviations: OR, Odd Ratio; 95% CI, 95% Confidence Interval.

*** p<0.0001

** p<0.01

* p<0.05

^†^ p<0.1.

^a^ Models also included, predisposing, enabling, and behaviour-related factors. Full models are shown in S2 Table.

^b^ Age was centered at the mean of the distribution in 1994/95 (39 years). All models also included a quadratic age term

^c^ Estimates are variances.

As with CAM use, we included predisposing, enabling, and behavior-related factors to Model 2 (S2 Table, Model 2a). There were no significant differences in chiropractic use between men and women. Those with higher income, who were overweight or obese, current smokers, and physically active were more likely to consult with chiropractors. When these variables were considered, the estimates of the age and cohort effects were reduced but remained significant.

Model 3 in Table 4 shows the estimates for age, cohort, need factors, and period with the full model presented in S2 Table, Model 3. The estimates of age effects were slightly reduced, although they remained significant, while the cohort effect estimates were no longer significant. Lastly, Model 4 shows the results of adding the use of conventional care to Model 3. Although significant, the inclusion of use of conventional care did not alter the age and cohort estimates.

#### Role of need for care and use of conventional care

Findings from the fully adjusted model (Table 4, Model 4) indicate that need for care factors (i.e. chronic conditions and pain) were significantly associated with chiropractic use, such that having more chronic conditions and/or pain affecting activities were strong positive predictors of chiropractic use. Furthermore, the use of conventional care was a significant and strong predictor of chiropractic use. Those consulting with primary care physicians and/or with specialists had higher odds of consulting with chiropractors.

### Sub-analyses

Our models adjusting for attrition showed no significant differences in CAM use between those who died, dropped-out, or remained for the duration of the study. Estimates for the factors associated with CAM use were similar to those obtained in the main analyses. Further analyses restricted to those who remained for the duration of the study showed that cohort differences in CAM use and the relationships of the factors examined remained unchanged. These analyses did not alter our conclusions.

The analyses for use of massage therapy, acupuncture, and homeopathy/naturopathy separately showed similar patterns and predictors of use over time to those of all CAM use. Furthermore, results from the models including individual chronic conditions suggested that although back pain was the most common chronic condition reported associated with chiropractic use, cohort differences were not explain by cohort differences in back pain. Lastly, the analyses controlling for use of conventional medicine in the previous cycle of data collection yielded findings comparable to the main analyses. Results from these analyses are available upon request.

## Discussion

Using data from a large longitudinal national population survey spanning 16 years, this study examined CAM and chiropractic use among baby boomers, Gen Xers, and pre-boomers in the context of need for care (i.e. chronic conditions and pain) and the use of conventional care (i.e. visits to physicians). There were substantial cohort differences in CAM and chiropractic use, with each succeeding recent cohort reporting higher use of these practitioners (e.g. Gen Xers reported greater CAM use than younger boomers and so on). In addition to cohort differences there was an increase in CAM use, but not chiropractic use, over time (period effect) across all ages. Of interest was that different factors underlay cohort differences in CAM and chiropractic use. Cohort differences in CAM use were partly related to period effects with greater CAM use over time, whereas differences in chiropractic use were related to differences in need for care. The use of conventional care was positively related to greater use of CAM and chiropractic, but was not related to changes over time or cohort differences.

Higher CAM use over time, independent of changes in the individual factors examined, is in keeping with studies suggesting that the growing interest in CAM reflects societal changes that have been happening for several decades. These include the rise in medical consumerism, the self-care movement, and the resurgence of holistic health in the 1970s [17, 18, 47, 48]. In addition, many physicians are more engaged with CAM practices and therapies than previously, which may explain some of the findings. For example, a survey of Canadian primary care physicians found that 12% offered CAM services in their practice [49] and a literature review found that 40% of physicians referred patients to chiropractors for the management of chronic pain and back problems [50].

That chiropractic use remained relatively stable between 1994 through 2011 is in accord with two smaller studies focused on healthcare use in provinces within Canada [16, 51]. The trend is of interest given that there has been an increase in the number of chiropractors during this time period (15.9 vs. 24.3 per 100000 population in 1997 and 2011, respectively) [52]. The reasons why use of chiropractic services is not noticeably changing are unknown. It is possible that, as other CAM therapies and practices have become more widely accepted and used, as was found in these data, it has created competition from other healthcare providers [51, 53]. Future research would benefit from asking individuals directly about CAM preferences and choices in care.

In our study, although chronic conditions and pain were strongly associated with higher CAM use overall, the higher CAM use in Gen Xers and baby boomers was not related to cohort differences in these factors. Cohort differences in CAM use were partly related to period effects of increasing CAM use over time. As noted, there have been significant changes in healthcare consumers’ values and expectations that appear to have had an impact on how more recent cohorts approach their healthcare choices. The greater CAM use in Gen Xers and boomers may be because they have been exposed, from an early age, to alternative treatments as a more normalized part of the healthcare culture. It may also reflect that members of recent generations share beliefs that are align with the holistic principles of CAM towards healthcare. As such, these generations may use CAM not only for treatment purposes but for health promotion, supporting the idea that CAM is beneficial in maintaining well-being and preventing illness. It is important for health services researchers and policy makers to understand the reasons why individuals from different generations use CAM to develop appropriate policies. In contrast, although back pain was the most common chronic condition reported by those using chiropractic services, back pain alone did not explain cohort differences in chiropractic use. The greater number of chronic conditions in recent cohorts contributed to the greater chiropractic use in these cohorts.

Our finding that use of conventional care did not reduce CAM consumption align with previous research suggesting that CAM users do not abandon conventional care [11, 54, 55]. Some proponents of CAM therapies and practices have speculated that since CAM focuses on preventive care and is less expensive than conventional care, promoting the use of CAM may help control increasing healthcare costs [34, 56]. However, research in this area is too scant to inform policy decisions. Since in our study we found that CAM users not only use conventional care more frequently but they also use more services, widespread CAM use may not reduce healthcare costs in this context. As CAM is not covered by the provincial health plans in Canada, it is likely that growing CAM use will translate into increased out-of-pocket costs for CAM users. This joint use of conventional care and CAM is also important in light of studies showing that more than 50% of CAM users do not disclose their CAM use to their conventional healthcare providers [5, 57, 58]. Studies have shown that when patients disclose CAM use to their physicians they experience a better patient–physician relationship and improve quality of care [59, 60]. From a policy perspective, understanding more about the patterns of use of multiple healthcare services in the population is particularly important because the evidence base about the safety and efficacy of CAM is limited. Future research is warranted to distinguish those who use CAM for treatment and/or for health promotion, as this will have implications for determining whether, and how CAM or specific forms of CAM can be integrated within the current healthcare delivery system.

### Strengths and limitations

This study enhances the current literature by drawing upon panel data from a large population longitudinal survey, providing the most current and comprehensive data available in Canada describing CAM use. We were able to compare CAM use across different cohorts at the same chronological age over a period of 16 years. However, the study is not without limitations. The analyses focused on practitioner-based CAM use and did not include alternative therapies. As a result, it may under-represent CAM use as people may be using a wide range of alternative therapies (e.g. taking herbal supplements) without consulting CAM practitioners. Although the survey collected information on the presence of chronic conditions and pain, it did not link these conditions and CAM use. Consequently, we were unable to identify the specific conditions for which individuals consulted with CAM practitioners. Also, information is not available on all the factors that motivate individuals to consult with CAM practitioners. For example, it is unknown if seeking care from CAM practitioners was by referral from physicians, related to lifestyle and general health and well-being, or if the decision was motivated by disenchantment with the conventional healthcare system. Lastly, given the longitudinal nature of the study and the long follow-up time, almost two-fifths of the sample died or dropped-out during follow-up. However, we were able to examine the impact of these losses on the results and these did not change our conclusions.

The analyses presented in this paper did not use sample weights. Although it has been suggested that failing to account for the complex design in multilevel analyses can produce biased parameter estimates, using a single weight combining level-1 and level-2 sampling design elements––as is the case for the NPHS––can also produce bias results [61]. In keeping with this notion, a simulation study comparing different methods for incorporating sampling weights into multilevel models suggested that unless weights are included properly (e.g. properly re-scale weights at each level) in the estimation, the un-weighted analysis yielded results similar to those that accounted for the complex design. More specifically, the study found that overall weighted and un-weighted parameter estimates and standard errors were generally comparable [62]. Furthermore, we fit the two-level models for CAM and chiropractic use adjusting for the individual level predictors with and without weights. The findings from the weighted analyses were not appreciable different to those from the un-weighted analyses. Taken all these together we do not expect that the un-weighted analyses presented in this paper affected the results and conclusions substantially.

## Conclusions

Our study adds to the literature by examining the lifecourse trajectories of practitioner-based CAM use using longitudinal data from a large national population survey. The findings indicate that Gen Xers and younger and older boomers were more likely to consult with CAM practitioners than pre-boomers, and that CAM use, excluding chiropractors, has increased over time across all ages (period effect). We also found that CAM users are also users of conventional care. This underscores the importance of doctors asking their patients about their CAM use. Finally, the increasing trend of CAM use over time highlights the need for continuing efforts to rigorously evaluate the safety, mechanisms, and cost-effectiveness of CAM therapies and practices.

## Supporting information

S1 Table CAM use: Results from logistic growth models a. Canadian National Population Health Survey, 1994–2011
(DOCX)

S2 Table Chiropractic use: Results from logistic growth models a. Canadian National Population Health Survey, 1994–2011
(DOCX)
