# Supplementary material for: Changes in the use practitioner-based complementary and alternative medicine over time in Canada: Cohort and period effects
Source: PLoS One. 2017 May 11;12(5):e0177307. doi: 10.1371/journal.pone.0177307 (PMC5426710; doi:10.1371/journal.pone.0177307)
Supplement: S1 Table — Canadian National Population Health Survey, 1994–2011 (DOCX) [file pone.0177307.s001.docx]

CAM use: Results from logistic growth models ^a^. Canadian National Population Health Survey, 1994-2011

|  | **Model 2a** |  | **Model 3** |  | **Model 4** |
| --- | --- | --- | --- | --- | --- |
|  | **OR (95% CI)** |  | **OR (95% CI)** |  | **OR (95% CI)** |
| **Fixed effects** |  |  |  |  |  |
| ***Age and Cohort Effects*** |  |  |  |  |  |
| Linear age ^b^ | 1.03 (1.01;1.04)^***^ |  | 1.02 (1.00;1.03)^***^ |  | 1.07 (1.06;1.08)^***^ |
| Birth cohort |  |  |  |  |  |
| Generation X | 1.70 (1.02;3.00)^***^ |  | 1.78 (1.03;3.07)^***^ |  | 1.51 (1.14;2.00)^***^ |
| Younger Baby Boomer | 1.34 (0.85;2.10) |  | 1.36 (1.01;2.10)^**^ |  | 1.25 (1.01;1.61)^*^ |
| Older Baby Boomer | 1.17 (0.83;1.63) |  | 1.13 (0.99;1.57)^*^ |  | 1.12 (0.99;1.40) |
| World War II | 0.89 (0.70;1.13) |  | 0.87 (0.69;1.10) |  | 0.94 (0.77;1.15) |
| Pre-World War | 1.00 |  | 1.00 |  | 1.00 |
| ***Predisposing factors*** |  |  |  |  |  |
| Sex (Women vs. Men) | 2.11 (1.93;2.30) |  | 2.09 (1.91;2.28) ^***^ |  | 2.03 (1.86;2.22) ^***^ |
| Education |  |  |  |  |  |
| <12 years |  |  | 1.00 |  | 1.00 |
| 12-15 years | 1.54 (1.37;1.72) |  | 1.52 (1.35;1.70) ^***^ |  | 1.52 (1.35;1.70) ^***^ |
| 16+ years | 2.43 (2.12;2.78) |  | 2.39 (2.08;2.74) ^***^ |  | 2.36 (2.06;2.71) ^***^ |
| ***Enabling factors*** |  |  |  |  |  |
| Income quartiles |  |  |  |  |  |
| Bottom (Q1) |  |  | 1.00 |  | 1.00 |
| Q2 | 1.15 (1.04;1.27) |  | 1.14 (1.03;1.26) ^***^ |  | 1.15 (1.04;1.27) ^***^ |
| Q3 | 1.33 (1.20;1.47) |  | 1.29 (1.17;1.43) ^***^ |  | 1.32 (1.19;1.46) ^***^ |
| Top (Q4) | 1.65 (1.47;1.84) |  | 1.57 (1.41;1.76) ^***^ |  | 1.64 (1.47;1.83) ^***^ |
| Missing | 1.16 (0.95;1.43) |  | 1.11 (0.90;1.36) |  | 1.17 (0.95;1.44) |
| Regular source of care | 0.98 (0.89;1.09) |  | 0.99 (0.89;1.10) |  | 0.89 (0.80;0.99) ^*^ |
| ***Behavioral Risk Factors*** |  |  |  |  |  |
| Smoking status |  |  |  |  |  |
| Current | 1.30 (1.18;1.42) |  | 1.28 (1.17;1.40) ^***^ |  | 1.28 (1.17;1.40) ^***^ |
| Former | 1.09 (0.98;1.21) |  | 1.10 (0.99;1.22) |  | 1.09 (0.98;1.21) |
| Never |  |  | 1.00 |  | 1.00 |
| BMI |  |  |  |  |  |
| Underweight | 1.31 (0.98;1.76) |  | 1.34 (1.00;1.80) ^*^ |  | 1.31 (0.97;1.75) |
| Normal | 1.41 (1.20;1.65) |  | 1.44 (1.22;1.69) ^***^ |  | 1.42 (1.21;1.67) ^***^ |
| Overweight | 1.27 (1.08;1.49) |  | 1.28 (1.09;1.50) ^**^ |  | 1.28 (1.09;1.50) ^**^ |
| Moderate obese | 1.12 (0.95;1.31) |  | 1.12 (0.95;1.32) |  | 1.12 (0.95;1.32) |
| Severe obese |  |  | 1.00 |  | 1.00 |
| Physically active | 1.16 (1.09;1.24) |  | 1.15 (1.08;1.23) ^***^ |  | 1.17 (1.10;1.24) ^***^ |
| Sedentary lifestyle | 1.11 (1.03;1.19) |  | 1.10 (1.03;1.18) ^**^ |  | 1.10 (1.02;1.18) ^*^ |
| ***Need for Health Care*** |  |  |  |  |  |
| Chronic conditions |  |  |  |  |  |
| 2+ | 1.94 (1.77;2.12) |  | 1.91 (1.75;2.08)^***^ |  | 1.79 (1.64;1.96)^***^ |
| 1 | 1.46 (1.35;1.59) |  | 1.45 (1.34;1.58)^***^ |  | 1.40 (1.29;1.52)^***^ |
| None |  |  | 1.00 |  | 1.00 |
| Pain that prevents activity | 1.90 (1.74;2.07) |  | 1.90 (1.74;2.10)^***^ |  | 1.81 (1.66;1.98)^***^ |
| ***Use of Conventional Care*** |  |  |  |  |  |
| Physician visits |  |  |  |  |  |
| Both |  |  |  |  | 1.78 (1.60;1.97)^***^ |
| Primary Care Only |  |  |  |  | 1.44 (1.30;1.58)^***^ |
| Specialists Only |  |  |  |  | 1.21 (1.00;1.47)^*^ |
| No visits |  |  |  |  | 1.00 |
| **Random effects ^c^** |  |  |  |  |  |
| Period | 0.15 (0.03;0.25)^***^ |  | 0.11 (0.01;0.20)^***^ |  | 0.11 (0.02;0.22)^***^ |
| Individual | 1.99 (1.78;2.10)^***^ |  | 1.92 (1.82;2.02)^***^ |  | 1.91 (1.81;2.01)^***^ |

CAM, Complementary and Alternative Medicine; OR, Odd Ratio; 95% CI, 95% Confidence Interval.

*^***^ p<0.0001, ^**^ p<0.01, ^*^ p<0.05, ^†^ p<0.1*.

^a^ Cross-classified random intercept model.

^b^ Age is centered at the mean of the distribution in 1994 (39 years). All models also included a quadratic age term.

^c^ Estimates are variances.
