# Supplementary material for: Changes in the use practitioner-based complementary and alternative medicine over time in Canada: Cohort and period effects
Source: PLoS One. 2017 May 11;12(5):e0177307. doi: 10.1371/journal.pone.0177307 (PMC5426710; doi:10.1371/journal.pone.0177307)
Supplement: S2 Table — Canadian National Population Health Survey, 1994–2011 (DOCX) [file pone.0177307.s002.docx]

Chiropractic use: Results from logistic growth models ^a^. Canadian National Population Health Survey, 1994-2011

|  | **Model 2a** |  | **Model 3** |  | **Model 4** |
| --- | --- | --- | --- | --- | --- |
|  | **OR (95% CI)** |  | **OR (95% CI)** |  | **OR (95% CI)** |
| **Fixed effects** |  |  |  |  |  |
| ***Age and Cohort Effects*** |  |  |  |  |  |
| Linear age ^b^ | 1.02 (1.02;1.03) ^***^ |  | 1.01 (1.01;1.02) ^***^ |  | 1.01 (1.01;1.02) ^***^ |
| Birth cohort |  |  |  |  |  |
| Generation X | 1.48 (1.13;1.94) ^***^ |  | 1.12 (0.85;1.46) |  | 1.15 (0.88;1.51) |
| Younger Baby Boomer | 1.26 (0.99;1.60) |  | 1.02 (0.80;1.29) |  | 1.04 (0.82;1.32) |
| Older Baby Boomer | 1.03 (0.83;1.27) |  | 0.87 (0.70;1.08) |  | 0.88 (0.71;1.09) |
| World War II | 0.92 (0.76;1.12) |  | 0.83 (0.68;1.01) |  | 0.83 (0.69;1.01) |
| Pre-World War | 1.00 |  | 1.00 |  | 1.00 |
| ***Predisposing factors*** |  |  |  |  |  |
| Sex (Women vs. Men) | 1.10 (1.00;1.20) ^*^ |  | 1.00 (0.91;1.10) |  | 0.99 (0.90;1.08) |
| Education |  |  |  |  |  |
| <12 years | 1.00 |  | 1.00 |  | 1.00 |
| 12-15 years | 1.23 (1.10;1.38) ^***^ |  | 1.23 (1.10;1.38) ^***^ |  | 1.23 (1.10;1.37) ^***^ |
| 16+ years | 1.14 (1.01;1.32) ^*^ |  | 1.15 (1.01;1.32) ^*^ |  | 1.14 (1.02;1.31) ^*^ |
| ***Enabling factors*** |  |  |  |  |  |
| Income quartiles |  |  |  |  |  |
| Bottom (Q1) | 1.00 |  | 1.00 |  | 1.00 |
| Q2 | 1.09 (0.99;1.20) |  | 1.14 (1.03;1.25) ^**^ |  | 1.14 (1.04;1.25) ^**^ |
| Q3 | 1.27 (1.15;1.39) ^***^ |  | 1.32 (1.20;1.45) ^***^ |  | 1.32 (1.20;1.45) ^***^ |
| Top (Q4) | 1.35 (1.21;1.50) ^***^ |  | 1.43 (1.28;1.60) ^***^ |  | 1.43 (1.28;1.59) ^***^ |
| Missing | 0.95 (0.78;1.17) |  | 0.99 (0.81;1.22) |  | 0.99 (0.81;1.22) |
| Regular source of care | 1.22 (1.10;1.34) ^***^ |  | 1.13 (1.02;1.26) ^***^ |  | 1.08 (0.98;1.20) |
| ***Behavioral Risk Factors*** |  |  |  |  |  |
| Smoking status |  |  |  |  |  |
| Current | 1.25 (1.14;1.37) ^***^ |  | 1.28 (1.16;1.40) ^***^ |  | 1.27 (1.16;1.39) ^***^ |
| Former | 1.04 (0.93;1.15) |  | 1.09 (0.98;1.22) |  | 1.09 (0.98;1.21) |
| Never | 1.00 |  | 1.00 |  | 1.00 |
| BMI |  |  |  |  |  |
| Underweight | 1.08 (0.92;1.28) |  | 1.09 (0.93;1.53) |  | 1.10 (0.98;1.53) |
| Normal | 0.83 (0.60;1.14) |  | 0.99 (0.72;1.36) |  | 0.99 (0.72;1.37) |
| Overweight | 1.13 (0.96;1.33) |  | 1.30 (1.10;1.53) ^***^ |  | 1.30 (1.11;1.54) ^***^ |
| Moderate obese | 1.12 (0.95;1.32) |  | 1.21 (1.03;1.43) ^**^ |  | 1.21 (1.03;1.43) ^**^ |
| Severe obese |  |  |  |  |  |
| Physically active | 1.09 (1.03;1.16) ^**^ |  | 1.10 (1.03;1.17) ^**^ |  | 1.10 (1.03;1.17) ^**^ |
| Sedentary lifestyle | 1.00 (0.93;1.07) |  | 0.96 (0.90;1.04) |  | 0.96 (0.89;1.03) |
| ***Need for Health Care*** |  |  |  |  |  |
| Chronic conditions |  |  |  |  |  |
| 2+ |  |  | 2.37 (2.17;2.58) ^***^ |  | 2.31 (2.11;2.52) ^***^ |
| 1 |  |  | 1.61 (1.49;1.75) ^***^ |  | 1.58 (1.46;1.72) ^***^ |
| None |  |  | 1.00 |  | 1.00 |
| Pain prevents activity |  |  | 1.44 (1.32;1.58) ^***^ |  | 1.43 (1.31;1.57) ^***^ |
| ***Use of Conventional Care*** |  |  |  |  |  |
| Physician visits |  |  |  |  |  |
| Both |  |  |  |  | 1.22 (1.11;1.35) ^***^ |
| Primary Care Only |  |  |  |  | 1.25 (1.14;1.36) ^***^ |
| Specialists Only |  |  |  |  | 1.01 (0.84;1.23) |
| No visits |  |  |  |  | 1.00 |
| **Random effects ^c^** |  |  |  |  |  |
| Period | 0.01 (0.00;0.03) |  | 0.01 (0.00;0.03) |  | 0.01 (0.00;0.04) |
| Individual | 2.63 (2.51;2.75) ^***^ |  | 2.59 (2.47;2.71) ^***^ |  | 2.59 (2.47;2.70) ^***^ |

OR, Odd Ratio; 95% CI, 95% Confidence Interval.

*^***^ p<0.0001, ^**^ p<0.01, ^*^ p<0.05, ^†^ p<0.1*.

^a^ Cross-classified random intercept model.

^b^ Age is centered at the mean of the distribution in 1994 (39 years). All models also included a quadratic age term.

^c^ Estimates are variances.
